# Update and Evaluation of a High-Throughput In Vitro Mass Balance Distribution Model: IV-MBM EQP v2.0

**DOI:** 10.3390/toxics9110315

**Published:** 2021-11-20

**Authors:** James M. Armitage, Alessandro Sangion, Rohan Parmar, Alexandra B. Looky, Jon A. Arnot

**Affiliations:** 1 AES Armitage Environmental Sciences, Inc., Ottawa, ON K1L 8C3, Canada; 2 ARC Arnot Research and Consulting, Inc., Toronto, ON M4M 1W4, Canada; alessandro.sangion@mail.utoronto.ca (A.S.); rohanpurr@gmail.com (R.P.); alexandrablooky@gmail.com (A.B.L.); jon@arnotresearch.com (J.A.A.); 3 Department of Physical and Environmental Sciences, University of Toronto Scarborough, Toronto, ON M1C 1A4, Canada; 4 Department of Pharmacology and Toxicology, University of Toronto, Toronto, ON M5S 1A8, Canada

**Keywords:** in vitro, bioactivity/toxicity, distribution, Q-IVIVE

## Abstract

This study demonstrates the utility of an updated mass balance model for predicting the distribution of organic chemicals in in vitro test systems (IV-MBM EQP v2.0) and evaluates its performance with empirical data. The IV-MBM EQP v2.0 tool was parameterized and applied to four independent data sets with measured ratios of bulk medium or freely-dissolved to initial nominal concentrations (e.g., C24/C0 where C24 is the measured concentration after 24 h of exposure and C0 is the initial nominal concentration). Model performance varied depending on the data set, chemical properties (e.g., “volatiles” vs. “non-volatiles”, neutral vs. ionizable organics), and model assumptions but overall is deemed acceptable. For example, the r^2^ was greater than 0.8 and the mean absolute error (*MAE*) in the predictions was less than a factor of two for most neutral organics included. Model performance was not as good for the ionizable organic chemicals included but the r^2^ was still greater than 0.7 and the *MAE* less than a factor of three. The IV-MBM EQP v2.0 model was subsequently applied to several hundred chemicals on Canada’s Domestic Substances List (DSL) with nominal effects data (*AC*50s) reported for two in vitro assays. We report the frequency of chemicals with *AC*50s corresponding to predicted cell membrane concentrations in the baseline toxicity range (i.e., >20–60 mM) and tabulate the number of chemicals with “volatility issues” (majority of chemical in headspace) and “solubility issues” (freely-dissolved concentration greater than water solubility after distribution). In addition, the predicted “equivalent EQP blood concentrations” (i.e., blood concentration at equilibrium with predicted cellular concentration) were compared to the *AC*50s as a function of hydrophobicity (log octanol-water partition or distribution ratio). The predicted equivalent EQP blood concentrations exceed the *AC*50 by up to a factor of 100 depending on hydrophobicity and assay conditions. The implications of using *AC*50s as direct surrogates for human blood concentrations when estimating the oral equivalent doses using a toxicokinetic model (i.e., reverse dosimetry) are then briefly discussed.

## 1. Introduction

In vitro bioactivity and toxicity data are increasingly being reported in the scientific literature in response to the desire to reduce in vivo animal testing and reduce the amount of time and effort required to characterize the potential hazards and risks of the thousands of chemicals in commerce. For example, the ToxCast program (https://www.epa.gov/chemical-research/toxicity-forecasting) has generated in vitro data for thousands of chemicals across hundreds of different assays and assay endpoints [1,2].

A major challenge with the interpretation and application of in vitro toxicity test data is the typical practice of using nominal (administered) bulk medium concentrations to report the dose associated with responses instead of more directly relevant metrics such as the freely-dissolved or cellular concentration [3,4,5,6,7,8,9,10,11,12,13,14,15]. In some cases, the volume of medium in the well plate is too small to allow for experimental dose confirmation (e.g., the 1536 well plates used in some ToxCast assays). In other situations, the resources and expertise required to conduct experimental dose confirmations are unavailable. While in vitro bioactivity and toxicity data on a nominal concentration basis can and have been used to assess toxicity, the benefits of experimental dose confirmation or estimation of additional dose metrics for subsequent data interpretation and analyses are clear. For example, the (estimated) freely-dissolved concentrations from in vitro testing are expected to be more comparable to the (estimated) freely-dissolved concentrations corresponding to effects from in vivo testing and hence are more useful and reliable for in vitro-in vivo extrapolation (IVIVE) (e.g., [10,16]). To address the fact that experimental dose confirmation is not always feasible, various researchers have developed and applied mass balance models to simulate the distribution of organic chemicals in in vitro test systems [17,18,19,20,21,22,23,24]. Regression-based approaches to estimate depletion of the medium as a function of octanol-water partitioning and Henry’s Law constant have also been proposed [25]. The value of characterizing the disposition of organic chemicals in in vitro test systems using a mass balance model was recently demonstrated by Casey et al. [26] in their evaluation of IVIVE approaches relating in vitro estrogen receptor activity to in vivo estrogenic effects from rodent uterotrophic studies. Specifically, Casey et al. found that the predictive performance of their extrapolation approaches was significantly improved by adjusting in vitro bioactivity data using the cellular Enrichment Factor (EF, ratio of predicted cell concentration to nominal medium concentration) generated by the in vitro distribution model described by Armitage et al. [18].

The main objective of this work is to describe, apply and evaluate an updated version of the Armitage et al. in vitro mass balance model originally published in 2014 (“IV-MBM v1.0”) [18]. While the general utility of the 2014 version of the model was demonstrated in the related publication, the model has several limitations. For example, sorption to plastic was excluded in the mass balance calculations and only neutral forms of organic chemicals could be simulated. Furthermore, parameterization of the model to match the wide range of experimental conditions for a large number of in vitro test systems described in the literature was not intuitive. In addition, the original model was not rigorously evaluated due to the lack of suitable experimental data. A number of studies quantifying the in vitro disposition of organic chemicals have been published since 2014 which now permit model performance to be better assessed. The new version of the tool, IV-MBM EQP v2.0, its updates, novel outputs, and various model applications are documented herein. The potential implications for using in vitro bioactivity and toxicity data for interpretating relative hazard (i.e., potency) and for using in vitro data for reverse dosimetry and IVIVE, e.g., calculating oral equivalent doses (OEDs) and related metrics are also discussed.

## 2. Materials and Methods

### 2.1. Basic Model Description

The IV-MBM EQP v2.0 is a “static” (i.e., equilibrium partitioning based) model for simulating the distribution of organic chemicals in in vitro test systems. Once added to the test system via the medium, the chemical is subject to various transport (diffusion) and partitioning processes (e.g., volatilization, specific and non-specific interactions with medium lipids and proteins, sorption to well plate plastics, uptake into cells) that determine the distribution of the chemical and influence the dose-response. The distribution of the chemical in the test system is calculated by the IV-MBM EQP v2.0 model as a function of partitioning ratios and volumes or surface areas of the various phases included (e.g., medium, cells, serum lipids, and albumin, vessel wall) [18]. The model output can be considered to represent the ultimate fate of the chemical in the test system given the experimental conditions assuming no changes in system parameters and characteristics. The kinetics of uptake and distribution cannot be simulated using an equilibrium partitioning-based model nor can processes such as degradation/biotransformation and cell growth be explicitly represented.

There is a large variety of in vitro test systems in terms of well plate size (e.g., 6–1536 well plate), working volume of medium (1–1000 µL), medium characteristics (e.g., 0–20% serum), and cell properties and initial seeding density. In addition, chemicals spanning a very wide range of properties have been tested in in vitro systems. Accordingly, a flexible and transparent modelling approach is required to allow users to simulate the distribution of organic chemicals across exposure scenarios. To better address these issues, the IV-MBM EQP v2.0 model has been updated with the following features, (i) proximate composition of cells expanded to include storage lipids, membrane lipids, bulk proteins and water, (ii) vessel wall (plastic) added to the mass balance with two options to estimate partitioning, (iii) ability to enter biopartitioning data (e.g., membrane-water, serum albumin water partitioning) directly instead of relying on default model calculations based on basic physical-chemical partitioning data (e.g., octanol-water partition ratio), (iv) ability to simulate ionizable organic chemicals (IOCs, monoprotic acids and bases), (v) ability to distinguish between sorption to anionic phospholipids and neutral phospholipids for IOCs [27], (vi) preliminary approach to simulate lysosomal sequestration (“ion trapping”) for basic IOCs (e.g., [28,29]), and (vii) guidance to facilitate more realistic parameterization of well plate characteristics (e.g., surface areas, total well plate volumes, typical cell seeding densities and mass per cell). See the Supplementary Material (Section S1) for more complete details on the updates implemented in the IV-MBM EQP v2.0 model.

Because the IV-MBM EQP v2.0 model was updated to allow the simulation of IOCs, the core equation from the Armitage et al. model [18] now uses partition ratios for neutral organics and distribution ratios for IOCs. i.e.,

For Neutral Organics:

(1) $$C_{W}=\frac{M_{T}}{V_{W}+K_{AW}V_{A}+K_{SaW}V_{Sa}+K_{SlW}V_{Sl}+K_{DsW}V_{Ds}+K_{CW}V_{C}+K_{PlW}A_{Pl}}$$

where *M_T_* is the total amount of chemical added to the test system (µmol), *C_W_* is the predicted freely-dissolved concentration in the medium (µM), *V_W_* is the volume of medium (aqueous phase only) (µL), *K_AW_* is the dimensionless air-water partition ratio, *V_A_* is the volume of headspace, *K_SaW_* is the serum albumin-water partition ratio, *V_SaW_* is the volume of serum albumin, *K_SlW_* is the storage lipid-water partition ratio, *V_Sl_* is the volume of storage lipid, *K_DsW_* is the “other dissolved organic matter”-water partition ratio, *V_Ds_* is the volume of “other dissolved organic matter”, *K_CW_* is the cell-water partition ratio, *V_C_* is the volume of cells and *K_PlW_* is the plastic-water partition ratio, *A_Pl_* is the surface area of plastic exposed to the medium.

Once the freely-dissolved concentration in the medium is determined, concentrations and masses in other phases can be calculated using the corresponding partitioning ratios (e.g., cell concentration = *K_CW_* × *C_W_* ) and volumes (e.g., mass absorbed by cells = *K_CW_* × *C_W_* × *V_C_*).

For Ionizable Organic Chemicals (IOCs):

(2) $$C_{W}=\frac{M_{T}}{V_{W}+D_{AW}V_{A}+D_{SaW}V_{Sa}+D_{SlW}V_{Sl}+D_{DsW}V_{Ds}+D_{CW}V_{C}+D_{PlW}A_{Pl}}$$

where the partition ratios in Equation (1) are replaced with *pH*-dependent distribution ratios [30]. The user can specify the *pH* of the medium and the cell interior (cytoplasm). The *pH* of fluid within lysosomes can also be specified by the user as part of the preliminary approach to address lysosomal sequestration (“ion trapping”). Distribution ratios are calculated as a weighted average of the partition ratios of the neutral and charged form depending on *pH* and *pKa*. Partitioning data for neutral and charged forms can be entered by the user. In the absence of data for the charged form, partitioning is scaled to the values for the neutral form as described in the Supplementary Material (Section S1).

Note that the cell-water distribution ratio *D_CW_* in Equation (2) is adjusted to account for *pH* differences between the two aqueous phases using the following equations:

For acidic IOCs:

(3) $$D_{CW}^{Adj}=D_{CW}\cdot 10^{\left(Cell\;pH-Bulk\;pH\right)}$$

For basic IOCs:

(4) $$D_{CW}^{Adj}=D_{CW}\cdot 10^{\left(Bulk\;pH-Cell\;pH\right)}$$

Following this approach, the adjusted cell-water distribution ratio for acidic IOCs is 0.4-fold smaller if the cell *pH* = 7.0 and medium *pH* is 7.4; conversely, the adjusted cell-water distribution ratio for basic IOCs is 2.5-fold greater under these conditions. Note that strictly speaking the approach for IOCs described above can only be applied to monoprotic acids and bases. Multiprotic IOCs can be simulated by the IV-MBM EQP v2.0 model but the calculations can only consider the strongest acidic or basic functional group (i.e., the smallest acidic *pKa* or largest basic *pKa*).

### 2.2. Sorption to Vessel Wall (Plastic)

Sorption to the vessel wall of the well plate is calculated based on the surface area of plastic exposed to medium and plastic-water partition (or distribution) ratios. The user can enter their own estimates or select from two estimation approaches, the *K_OW_*-based single parameter linear free energy relationship (spLFER) derived by (i) Kramer [14] or (ii) Fischer et al. [31].

Kramer spLFER [14]:

(5) $$log\;K_{PlW}=0.97\;logK_{OW,N}-6.94$$

Fischer et al. spLFER [31]:

(6) $$log\;K_{PlW}=0.56\;logK_{OW,N}-4.64$$

These partitioning ratios are both in units of m^3^/m^2^ and apply to neutral chemicals only. The partitioning ratio of the charged form of an IOC is calculated from the value for the neutral form using a scaling factor (See the Supplementary Material, Section S1). Because the slopes and y-intercepts differ substantially, the two spLFERs are compared across a range of *log K_OW_* in the Supplementary Material (Section S2). A third spLFER derived by Fischer et al. [31] using sorption data over a shorter experimental duration is also considered. Predicted *log K_PlW_* values are within a factor of approximately 5 for chemicals with *log K_OW,N_* between 4.0 and 7.5 but differ more substantially (i.e., a factor of 5 or greater) for chemicals outside this range. These differences are expected to be most relevant for predicting the distribution of chemicals with *log K_OW_* values between 2 to 4 or *log K_OW,N_* greater than 7.5 particularly in in vitro test systems lacking fetal bovine serum (FBS). The ratio of medium volume to the surface area of plastic in contact with the aqueous phase may also influence model performance across different test systems for chemicals where predicted *log K_PlW_* are substantially different.

### 2.3. Depletion Factors (DF), Enrichment Factors (EF), Equivalent EQP Blood Concentrations

Depletion and Enrichment Factors were defined in Armitage et al. [18] and then presented as a function of hydrophobicity in the original publication for a set of chemicals with diverse partitioning properties. For convenience, the Depletion Factor (DF) and Enrichment Factor (EF) are given again below.

(7) $$DF={\left(\frac{C_{W}}{C_{N,I}}\right)}^{-1}$$

(8) $$EF=\frac{C_{C}}{C_{N,I}}$$

where *C_W_* is the predicted freely-dissolved concentration in the test medium, *C_N,I_* is the initial nominal medium concentration (e.g., IC50, EC50, or *AC*50) and *C_C_* is the predicted cellular concentration. *DF* quantifies the extent to which the freely-dissolved concentration is reduced in comparison to the nominal and is basically just the inverse of the concentration ratios used to evaluate the model (i.e., C24/C0). *EF* quantifies the extent to which the cellular concentration is enriched relative to the initial nominal medium concentration and is a more appropriate dose metric for extrapolation.

For high-throughput reverse dosimetry exercises (e.g., [32]), in vitro bioactivity data on a nominal concentration basis (e.g., *AC*50s) haven traditionally been divided by a predicted blood concentration (*C_SS_*) to arrive at an oral equivalent dose (*OED*, mg/kg/d), i.e.,

(9) $$OED=AC50\cdot \frac{1\frac{\mathrm{mg}}{\mathrm{kg}}/d}{C_{SS}}$$

This extrapolation method assumes that the *AC*50 is a direct surrogate for blood even though in vitro test media can vary widely in composition (e.g., FBS volume fraction from 0 to 20%) and hence the conditions in the in vitro environment are almost never identical to blood.

To address the potential bias inherent to this assumption, the IV-MBM EQP v2.0 tool outputs a novel metric called an equivalent EQP blood concentration (*EQP C_B_*):

(10) $$EQP\;C_{B}=\frac{K_{BW}}{K_{CW}}C_{C}\;or\;\frac{D_{BW}}{D_{CW}}C_{C}$$

where *K_CW_* or *D_CW_* is the cell-water partition or distribution ratio and *K_BW_* or *D_BW_* is the blood-water partition or distribution ratio. *EQP C_B_* is the blood concentration necessary to achieve the same chemical activity (or fugacity) as the predicted cell concentration from the in vitro test under equilibrium conditions. The estimated *EQP C_B_* can subsequently be related to the nominal *AC*50 to arrive at an Enrichment Factor similar to the *EF* defined above but using blood as the phase of interest, i.e.,

(11) $$EF_{B}=\frac{EQP\;C_{B}}{AC50}$$

*EF_B_* values of one indicate that *AC*50 is a good surrogate for the equivalent blood concentration whereas larger values quantify the extent to which the *AC*50 underestimates the equivalent concentration eliciting the response in cells from the in vitro test.

The blood-water partition or distribution ratios are calculated using biopartitioning estimates and the proximate composition suggested by Endo et al. [33] (See Supplementary Material, Section S3). Note that this calculation can also be made referenced to plasma (i.e., EF_P_) instead of blood. That output is included in the IV-MBM EQP v2.0 tool but not presented here as part of the illustrative model application.

### 2.4. Model Implementation (Excel/VBA)

The IV-MBM EQP v2.0 model is implemented as an Excel/VBA spreadsheet tool. The user navigates through a series of input sheets (Chemical Data, Options for Generic Calculations, Well plate characteristics, System Parameters) before generating output by clicking on a command button (ActiveX control). Macros must be enabled in the software for the model to function. The model can be obtained by request from the following website: https://arnotresearch.com/models/.

The minimal physical-chemical property inputs required to run the model are (i) molecular weight (*MW*, g/mol), (ii) melting point (*MP*, °C), (iii) IOC type and *pKa* (acids, bases), (iv) octanol-water partition ratio of the neutral form (*log K_OW,N_*), (v) air-water partition ratio of the neutral form (*log K_AW,N_*), and (vi) water solubility (mg/L). The user is also required to enter the effects concentration in units of µM.

The output generated by the model has displayed on a series of sheets including the following, (i) concentrations in all phases (i.e., cells, freely-dissolved medium, headspace, sorbed to serum albumin and lipids, sorbed to plastic), (ii) mass fractions in all phases, (iii) masses in all phases, (iv) the IVIVE related outputs and (v) partitioning data adjusted to the temperature, *pH* and ionic strength of the medium. The IV-MBM EQP v2.0 model also provides a “QA-QC” output sheet which documents the frequency of chemicals with the “volatility issue” (2/3 of chemical mass in headspace), “solubility issue” (freely-dissolved concentration > water solubility after distribution), and predicted cellular or membrane concentrations in the range of baseline toxicity (e.g., membrane concentrations greater than 20–60 mM) [34].

### 2.5. Model Implementation (R/EAS-E Suite)

The IV-MBM EQP v2.0 tool has also been coded in the R programming environment [35] and can be used in the freely accessible online Exposure And Safety Estimation (EAS-E) Suite platform (https://arnotresearch.com/eas-e-suite/). The same minimal inputs are required as for the Excel/VBA version and the R/EAS-E Suite implementation provides the same outputs. The main advantage is that this version of the IV-MBM EQP v2.0 tool is directly linked to the EAS-E Suite database, and physical-chemical properties required to parameterize the model for 50,000 discrete organic chemicals are provided automatically by the system based on user input of chemical CAS RN, name, or SMILES notation. This greatly facilitates the initial parameterization and application of the model. Users also have the option of including model input values they may prefer to the values provided in the EAS-E Suite platform (e.g., *log K_OW_*). Users are required to obtain *pKa* estimates for IOCs from available databases and/or estimation software and enter them manually. The EAS-E Suite implementation includes automated system parameterization for four specific cell lines (MCF7, HCT116, HEK293T, and HepG2) [20,36,37] in addition to a default generic cell. Whereas the Excel/VBA version allows the user to conduct simulations for multiple chemicals simultaneously (i.e., batch mode), the IV-MBM EQP v2.0 model currently implemented in EAS-E Suite can only conduct simulations for one chemical at a time.

### 2.6. Model Parameterization and Evaluation

The IV-MBM EQP v2.0 model was evaluated against four independent data sets [38,39,40,41] reporting either the ratio of bulk medium or freely-dissolved concentration to initial nominal after 24 or 96 h of exposure (e.g., C24h/C0). Note that three of these data sets were used recently to evaluate the 2014 version of IV-MBM by other researchers [25]. Some key features of these data sets are summarized in Table 1.

The IV-MBM EQP v2.0 model was parameterized using the physical-chemical property data reported in the Supporting Information of Stadnicka-Michalak et al. [25] for three data sets [38,39,41] and in the main text and Supporting Information of Huchthausen et al. [40]. The membrane-water and serum albumin-water partitioning data reported by Huchthausen et al. [40] were used to parameterize the model for that application. For the three other applications, partition and distribution ratios were estimated using the spLFERs and scaling factors implemented in the model (e.g., [31,42,43]). Parameterization of the test system (e.g., temperature, FBS volume fraction) was based on information provided in the various studies. Cell properties were also taken from the original studies and supplemented with data published elsewhere as necessary (e.g., [20,37,44]). Simulations were conducted using both options for plastic-water partitioning. See the Supplementary Material (Section S4) for additional details.

The bulk medium or freely-dissolved concentration ratios (e.g., C24/C0) were taken from the Supporting Information of Stadnicka-Michalak et al. [25] or calculated based on information provided in the original studies. The initial nominal medium concentrations were based on dosing information reported in each study and are as follows, (i) ‘metabolic activity’ EC50s at t = 0 for Tanneberger et al. [38], (ii) measured concentrations at t = 0 for Dupraz et al. [39], (iii) nominal IC10s for cytotoxicity or activation for Huchthausen et al. [40] and (iv) the lowest and highest nominal concentrations for Schug et al. [41].

In the case of the high dose from Schug et al. [41], the initial nominal medium concentrations exceed or equal the water solubilities reported in the study for 9 of the 16 chemicals. In the case of the low dose, the initial nominal medium concentration equals the water solubility for 1 of the 16 chemicals. While dosing up to saturation is discussed in the OECD guidance document on aqueous phase aquatic toxicity testing of difficult test chemicals [45], some of the high doses are well above saturation (e.g., 40-fold greater than water solubility reported for Muscenone^®^ delta). Following from expectations based on log linear solubility in the presence of cosolvents [46,47,48], the presence of DMSO at 0.5% (*v*/*v*) is not likely to be sufficient to solubilize the chemical for some of the high dose experiments. In any case, the dosing regime applied by Schug et al. is a complication from both an experimental and modelling perspective for many of the test chemicals. For example, the measured EC50s for Cachalox^®^ and Muscenone^®^ delta presented in the Supporting Information of Schug et al. [41] are approximately 5-fold greater than the reported water solubilities. A plausible explanation for these findings is that the test medium sampled from the wells and used for chemical analysis contained undissolved (i.e., pure phase) chemicals.

#### 2.6.1. Parameterization of Head Space Volume (*V_A_*)

A perceived limitation of the equilibrium partitioning-based mass balance approach is that volatilization from the test medium cannot be adequately accounted for. While it is true that volatilization cannot be treated as a kinetic process in the IV-MBM EQP v2.0 tool, the inclusion of headspace as a compartment in the model allows for the chemical loss out of the exposure medium. The possibility of significant sorption to adhesive materials covering the top of the well plates has also been raised [13,24]. The challenge is to estimate an appropriate volume of headspace to represent the test conditions and exposure duration. The minimum volume of headspace is the difference between the total volume of the well plate and the volume of the test medium added. If *V_A_* is defined this way, it implies that the well is perfectly sealed and gas exchange with the surrounding air outside of the well is not possible. Sorption to adhesive coverings is ignored as well. Increasing the volume of headspace beyond the minimum value implies that gas exchange can occur either because the well plate is left open or because leakage/gas exchange can occur. Although clearly a simplification of reality, an enlarged volume of headspace can also be viewed as a proxy for all three considerations (i.e., volatilization from medium into headspace, leakage to surrounding air, sorption to an adhesive covering). These assumptions are most relevant for relatively volatile chemicals with large air-water partition or distribution ratios (*K_AW_, D_AW_*). Note that we have also developed a time-variant version of the IV-MBM tool which simulates volatilization explicitly as a kinetic process that will be described and evaluated in a separate publication.

Simulations of the Tanneberger et al. [38] data were conducted under four assumptions regarding the volume of headspace (*V_A_*), (i) the minimal value of *V_A_*, (ii) an HS multiplier of 10, (iii) an HS multiplier of 50, and (iv) an HS multiplier of 100. This approach was taken because the chemicals included in this study span a wide range of air-water partition ratios including some relatively volatile chemicals. Simulations of the three other studies were also conducted across a range of assumptions regarding headspace volume but the evaluation focuses on the results obtained using the minimal value for *V_A_*.

#### 2.6.2. Metrics of Model Performance (*MB* and *MAE*)

The performance of the model was assessed using model bias (*MB*), mean absolute error (*MAE*), and by calculating the coefficient of determination (r^2^). *MB* and *MAE* are calculated as shown below.

(12) $$MB=\frac{\sum^{\;}log\frac{P}{O}}{n}$$

(13) $$MAE=\frac{\sum^{\;}ABS\left(log\frac{P}{O}\right)}{n}$$

where *P* is a predicted value, *O* is an observed value and n is the number of comparisons. For ease of interpretation, *MB* and *MAE* are also expressed as Factors of Agreement (FoA) i.e., 10*^MB^* and 10*^MAE^*. Coefficients of determination (r^2^) are calculated following the standard approach.

### 2.7. Illustrative Model Application (ToxCast Assays/EF_B_)

The IV-MBM EQP v2.0 tool was parameterized to match the test conditions of two ToxCast assays, AEID 767 (TOX21_Aromatase_Inhibition) and AEID 1325 (TOX21_p53_BLA_p4_ratio). Both tests are conducted in 1536 well plates but vary in the volume fraction of FBS present in the medium (0.5% and 10%). The details of the model parameterizations are documented in the Supplementary Material (Section S5).

Physical-chemical property data were compiled for chemicals on Canada’s Domestic Substances List (DSL) with reported *AC*50s (“Active Hits”). The model was then applied to simulate the distribution of the DSL chemicals in both assays with a focus on the estimated EQP blood concentrations and enrichment factors (*EF_B_*) described above. The frequencies of chemicals with the “volatility issue” (2/3 of chemical mass in headspace), “solubility issue” (freely-dissolved concentration > water solubility after distribution), and predicted cellular or membrane concentrations in the range of baseline toxicity (e.g., membrane concentrations greater than 20–60 mM) [34] are also tabulated and presented below.

## 3. Results

### 3.1. Model Performance for the Tanneberger et al. Data Set

**Figure 1:**
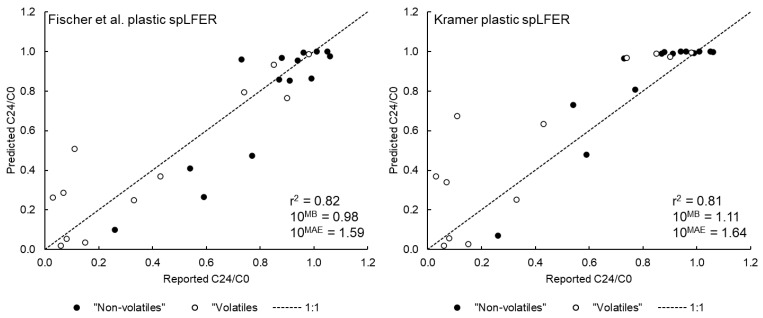
Model performance for the updated IV-MBM EQP v2.0 tool using Tanneberger et al. [38] dataset assuming a 50-fold larger effective volume of headspace and the two spLFERs for sorption to plastic.

The model performance of the IV-MBM EQP v2.0 for the Tanneberger et al. [38] data set is summarized in Table 2 and Figure 1. One chemical (permethrin, *log K_OW_* = 6.5, water solubility = 0.006 mg/L) was excluded from this evaluation set because the medium was predicted to be supersaturated (i.e., water solubility exceeded and pure phase chemical expected) at the nominal EC50 reported (9 mg/L) even after redistribution of the administered dose to various phases in the test system.

As shown in Table 2, the performance of the IV-MBM EQP v2.0 tool for the “non-volatiles” (i.e., *log K_AW_* or *log D_AW_* < −4.0), is satisfactory assuming the minimal headspace volume (*V_A_*). For both assumptions for plastic-water partitioning, the r^2^ is greater than 0.80 and the *MAE* corresponds to a Factor of Agreement (*FoA*) of approximately 1.3. The performance of the tool for the “volatiles” is poorer under the assumption of minimal *V_A_* but improves greatly as the volume of headspace is increased. The r^2^ assuming an HS multiplier of 50 and 100 exceed 0.80 and the *MAE* corresponds to *FoA* of less than two. These results suggest that leakage into surrounding air and/or sorption to the well plate covering occurred to a substantial extent during the course of the experiment for the “volatile” chemicals. Model performance is similar across the two assumptions regarding sorption to plastic. These results partly reflect the fact that the majority of the test chemicals are either relatively hydrophilic (*log K_OW,N_* < 2) or fall within the *log K_OW,N_* range where predicted *log K_PlW_* are relatively similar. Given the uncertainties inherent to both the model parameterization and measurements, the application of the IV-MBM EQP v2.0 tool to simulate both “non-volatiles” and “volatiles” is deemed acceptable. In particular, it is important to reiterate that no empirical biopartitioning estimates were used to parameterize the model and hence all calculations are based on the minimal set of inputs and the default approaches to estimate all required parameter values.

### 3.2. Model Performance for the Dupraz et al. and Huchthausen et al. Data Sets

**Figure 2:**
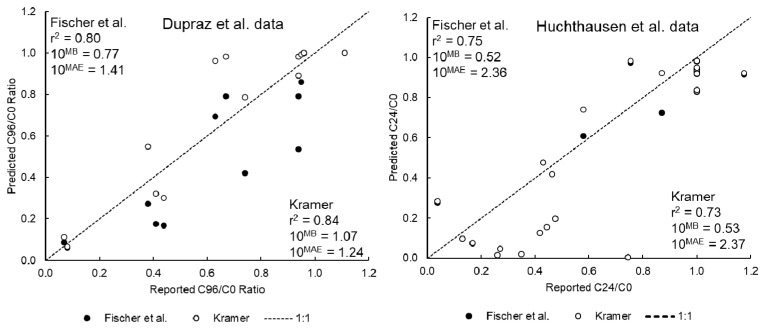
Model performance for the updated IV-MBM EQP v2.0 tool using Dupraz et al. [39] dataset (**left** panel, neutral organics) and Huchthausen et al. [40] dataset (**right** panel, IOCs) using the two spLFERs for sorption to plastic.

The performance of the IV-MBM EQP v2.0 tool for the Dupraz et al. [39] and Huchthausen et al. [40] datasets are presented in Figure 2. The performance for the Dupraz et al. [39] data is comparable to the performance for the “non-volatiles” from the Tanneberger et al. data set (i.e., r^2^ equal to or greater than 0.8 and *MAE* corresponding to an *FoA* of 1.5). As with the Tanneberger et al. application, model performance is similar using the Kramer spLFER or Fischer et al. spLFER to estimate plastic-water partitioning. These results again reflect the fact that the majority of the test chemicals are either hydrophilic (*log K_OW,N_* < 2) or fall within the *log K_OW,N_* range where predicted *log K_PlW_* are relatively similar.

The performance of the IV-MBM EQP v2.0 tool for the Huchthausen et al. [40] IOCs is not as good as for the neutral organics but the r^2^ is still greater than 0.7 and *MAE* corresponds to an *FoA* of approximately 2.5. Note that these simulations were conducted using the empirical membrane-water and serum albumin-water partitioning data reported by the authors in that publication. The performance of the model using the default spLFERs to estimate these parameters from octanol-water partitioning (i.e., *K_OW,N_* → *K_MW,N_* and *K_SaW,N_*) and scaling factors (e.g., *K_MW,N_* → *K_MW,I_*) is substantially worse (data not shown). These results speak to the challenges of estimating the sorption behavior of charged organic molecules using single parameter regression-based approaches, especially for serum albumin (see e.g., [40,49]). Even with empirical data for biopartitioning, there can be substantial discrepancies between the measurements and model predictions.

A good example of this is for naproxen (empirical *log D_MW_* = 2.17, empirical *log D_SaW_* = 5.21). The measured C24/C0 ratios (i.e., IC_10,free_/IC_10,nom_) for this compound are approximately 0.75 and 0.26 in the AREc32 and PPARγ test systems respectively whereas the predicted ratios are 0.003 and 0.02 respectively. Not only are the predicted ratios well below the reported values, the trend with FBS volume fraction is opposite. These observations are difficult to rationalize beyond speculating about the non-linear sorption behavior of this IOC in the test system. The discrepancy between modeled and measured C24/C0 ratios (IC_10,free_/IC_10,nom_) is likely driven by the relatively large value of the serum albumin distribution ratio which results in a large fraction of the chemical predicted to be sorbed to this phase. The implications for the extrapolation to equivalent EQP blood concentration or OED are unclear because the accuracy of the modeled cell concentrations cannot be ascertained. However, the lower freely-dissolved fraction (i.e., bioavailability) predicted by the model suggests that the extrapolated values would be conservatively assuming that the empirical freely-dissolved and cell concentrations and scale linearly and similarly to the predictions. Although the performance of the IV-MBM EQP v2.0 tool for IOCs is not as good as for neutral organics, the r^2^ and *MAE* are still reasonable for many compounds. Greater confidence in model output can be achieved using empirical biopartitioning data but clearly substantial uncertainties remain. More experimental data are needed but are unlikely to completely resolve the issues with simulating the more complex sorption behavior of IOCs in in vitro test systems.

### 3.3. Model Performance for the Schug et al. Data Set

**Figure 3:**
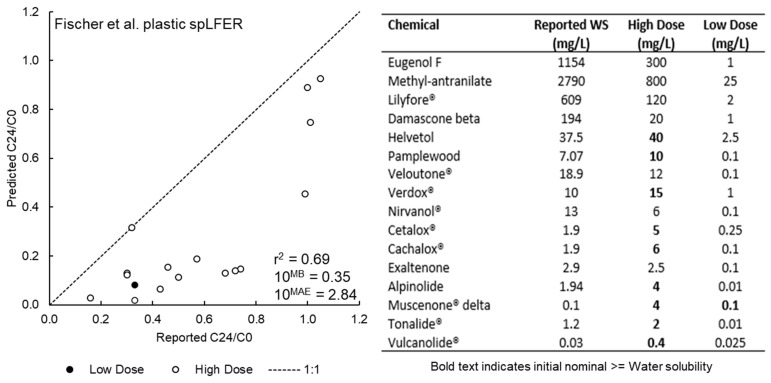
Model performance for the updated IV-MBM EQP v2.0 tool using Schug et al. [41] dataset for the “High” and “Low” Dose scenario (**left** panel) and dosing regime indicating the chemicals and instances of initial nominal concentrations exceeding the reported water solubilities (**right** panel).

The performance of the IV-MBM EQP v2.0 tool for the Schug et al. data set [41] is presented in Figure 3. As shown in Figure 3, the performance of the IV-MBM EQP v2.0 tool for the Schug et al. data is the worst of the four evaluations. For example, the r^2^ is below 0.7 and the *MAE* corresponds to a *FoA* approaching three regardless of model assumptions. The predicted C24/C0 ratios generally overestimate the degree of medium depletion (i.e., sorption to plastic and cells, volatilization) with the measurements indicating a greater fraction of chemical added remaining in the bulk medium. Model performance is similar for the “High Dose” and “Low Dose” scenarios and there are no significant differences in model output across the two assumptions for plastic-water partitioning. The amount of chemical predicted to be volatilized into the headspace does not explain the discrepancies for most of the instances where the model underestimates the C24/C0 ratio. For example, the predicted C24/C0 ratios for Cetalox^®^ and Cachalox^®^ across all HS multiplier scenarios are approximately 0.14 and 0.20 respectively whereas the reported ratios are 0.72 and 0.68 respectively. Formation of pure phase chemical (non-aqueous phase liquid or precipitates) and subsequent re-dissolution as the available freely-dissolved fraction is depleted via sorption to plastic and cells cannot be simulated by an EQP model. Since it is unclear at which dose the C24/C0 ratios were measured by Schug et al. and some of the empirical C24/C0 ratios exhibit inconsistent trends with partitioning properties (see Supplementary Material, Figure S1, Section S6), no further discussion of these results is included here. Initial doses above the water solubility limit are not ideal and the poorer performance of the IV-MBM EQP v2.0 model appears to be specific to this set of chemicals and experimental conditions.

### 3.4. Illustrative Model Application (Baseline Toxicity and EF_B_)

**Figure 4:**
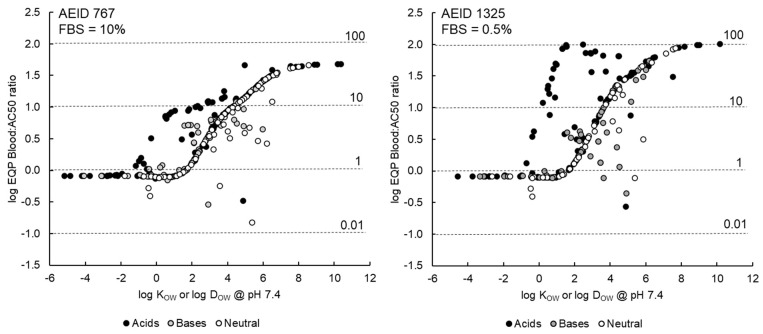
Log ratios of the predicted equivalent EQP blood concentrations and nominal *AC*50s (*log EF_B_*) for DSL chemicals with “Active Hits” in the ToxCast AEID 767 (*n* = 329, FBS = 10%, **left** panel) assay and ToxCast AEID 1325 assay (*n* = 217, FBS = 0.5%, **right** panel).

The number of DSL chemicals with *AC*50s in the AEID767 and AEID1325 ToxCast assays exhibiting the “volatility issue”, “solubility issue” and approaching the membrane concentration associated with baseline toxicity is summarized in Table 3. The predicted *EF_B_* as a function of hydrophobicity is presented in Figure 4.

As shown in Table 3, relatively few DSL compounds with reported *AC*50s for the AEID767 and AEID1325 assay exhibit the “volatility issue” (~1% or less) or “solubility issue” (~6.5% or less). The “solubility issue” occurs for more hydrophobic chemicals and is a familiar problem to those conducting aquatic toxicity testing with “difficult substances” (e.g., [45]). Cosolvents at the typical volume fraction (<1% *v*/*v*) are insufficient to solubilize the chemical once it is in the medium-cosolvent mixture, as can be predicted using log-linear solvation models. The influence of the presence of “pure phase” chemical on toxicity metrics is unclear but certainly, such instances should be subject to additional scrutiny.

Several in vivo and in vitro bioactivity/toxicity studies have demonstrated that membrane concentrations in the range of approximately 20–200 mM correspond with the baseline toxicity Mode of Action (MOA) (e.g., [34,50,51,52]). For in vitro testing, this phenomenon is also sometimes referred to as the “cytotoxic burst” [53]. The frequency with which the *AC*50s for the DSL chemicals in the AEID767 and AEID1325 assay correspond to membrane concentrations approaching the baseline toxicity range is relatively high. For example, approximately 40–45% and 23–28% of the *AC*50s correspond to a predicted membrane concentration greater than 20 mM and 60 mM respectively. The occurrence of baseline toxicity may be a true indicator of a chemical’s MOA or reflect a lack of sensitivity in the particular assay. Accordingly, some caution is required when interpreting these model outputs in the context of MOA. Nevertheless, the predicted membrane concentrations can still be used in a binary classification system (e.g., “apparent baseline toxicant” or “more potent MOA”) and included in a hazard assessment utilizing available in vivo toxicity data and other in silico lines of evidence.

As can be seen in Figure 4, there is a strong relationship between hydrophobicity and predicted equivalent EQP blood concentrations and Enrichment Factors (*EF_B_*). The *EF_B_s* approach a value of approximately 50 in the AEID767 assay (FBS = 10%) and 100 in the AEID1325 assay (FBS = 0.5%). The neutral organic chemicals follow a consistent trend with hydrophobicity with *EF_B_s* around 1 for chemicals with *log K_OW_* less than approximately two. The *EF_B_* increases steadily for chemicals with *log K_OW_* from 2–8 after which the upper plateau value is reached. Because we did not distinguish between acidic IOCs that are weakly and predominantly charged, the trend of *EF_B_* vs. hydrophobicity has two peaks. The weakly dissociated acidic IOCs follow the trend line for neutral organics whereas the peak *EF_B_* for the predominantly charged acidic IOCs is shifted to the left (i.e., occur at a lower *log D_OW_*@*pH* 7.4). The basic IOCs also include weakly and more strongly dissociated chemicals and therefore do not follow the same trend as the neutral organics. Note that some of the *EF_B_s* fall off the general trend line shown in Figure 4. These instances are for chemicals with the “solubility issue” i.e., freely-dissolved concentration above water solubility after distribution. These results may reflect the actual behavior of the chemical in the test system or errors in the water solubility estimate, many of which are predicted values.

#### Implications of Illustrative Model Application

The general implications for using nominal concentrations or model estimated concentrations for interpreting and applying bioactivity assay results are briefly summarized. For neutral organic chemicals with *log K_OW_* ≤ 2 or IOCs with *log D_OW_*@*pH*7.4 ≤ 0, the *EQP C_B_* and *AC*50s are comparable, and hence minimal bias is introduced to the reverse dosimetry exercise described above. As hydrophobicity increases, the *AC*50 increasingly underestimates the blood concentration consistent with the exposure where the bioactivity or toxicity is observed in the cells in in vitro test system. While the bias that occurs when using the administered (assumed) nominal concentration could be viewed as ‘conservative’ for risk-based prioritization exercises (i.e., resulting in a lower predicted *OED*), comparisons to in vivo toxicity data are confounded and therefore potentially misleading. Exploring the implications of this apparent bias is outside the scope of the current study but is considered a priority for future research.

## 4. Discussion

The main objective of this study was to describe, apply and evaluate the IV-MBM EQP v2.0 model to demonstrate its use for interpreting and applying in vitro bioactivity and toxicity data. As described above, the performance of the model is acceptable for “non-volatile” and “volatile” neutral organics if allowances for a leakage into the surrounding air and/or sorption to a well plate covering are included. Whereas the performance of the model for IOCs was not as good, the larger bias in model output reflects challenges with accurately parameterizing the model to capture more complex partitioning behaviors. Considering the amount of information provided by the IV-MBM EQP v2.0 model (e.g., concentrations, masses and mass fractions in all phases, frequency of “solubility” and “volatilization” issues), the ability to rapidly assess multiple chemicals (i.e., batch mode) and its relatively simple user interface in the Excel-based or EAS-E Suite implementations, more routine use of the IV-MBM tool is recommended. Depending on availability and suitability, any of the other in vitro distribution models published in the literature could also be considered. See Proença et al. [24] for a recent review and comparison of features and functionalities of the various tools.

Besides retrospective analyses of available in vitro toxicity data, it is also important to recognize that the IV-MBM EQP v2.0 model can be used prospectively to support the selection of appropriate doses for testing purposes (i.e., “in silico range finding”). For example, the potential for “solubility issues” to occur during testing of more hydrophobic chemicals could be reduced by simulating the distribution of the chemical across the proposed dosing regime to quantify when water solubilities are predicted to be exceeded. Because all key characteristics of the test system (e.g., well plate size, volume of medium, cell seeding density, FBS volume fraction) can readily be changed by the user, the IV-MBM EQP v2.0 model is extremely valuable when designing in vitro bioactivity or toxicity testing. Using the IV-MBM EQP v2.0 model to support experimental design could substantially improve data quality and save resources (time and money).

The illustrative model application has also demonstrated the potential utility of the IV-MBM EQP v2.0 model for MOA classification, reverse dosimetry, and other IVIVE applications of interest to the regulatory and scientific community. Given that in vitro data are expected to be increasingly relied upon in the future and experimental dose confirmation is rarely feasible, the benefits of applying mass balance models should be recognized and tools such as the IV-MBM EQP v2.0 must be freely available to facilitate their use more broadly across the scientific and regulatory community. Note that quantitative IVIVE can be conducted using several of the dose metrics generated by the IV-MBM EQP model (e.g., freely-dissolved concentration, cellular concentration, cellular enrichment factor, equivalent EQP blood concentration). The key requirement for comparisons to in vivo data is that both response metrics are expressed on a similar basis (i.e., “apples to apples”).

Although the results of the model evaluation for the four data sets included herein are encouraging, it is important to recognize and reiterate the limitations of the IV-MBM EQP v2.0 model which fall broadly into two main categories, (i) model formulation/applicability domain and (ii) data availability for model parameterization. The IV-MBM EQP v2.0 model assumes that instantaneous equilibrium (i.e., equivalent chemical activity) is achieved between all phases included in the mass balance and can only be applied to single-dose scenarios. Measurements using neutral organic chemicals across a range of hydrophobicity indicate that equilibrium is approached within 24 h (e.g., [54]) and hence this concern may only be relevant for short duration tests (1–6 h). Cellular uptake of predominantly charged IOCs and quaternary ammonium compounds is slower than for neutral organics and may require more time to approach equilibrium however.

The bioactivity or toxicity elicited during the test is assumed to occur at the equilibrium concentration in the cell and hence responses that may occur at lower concentrations could be obscured. Degradation of the chemical in the test medium or biotransformation by cells cannot be accounted for in the calculations and hence the model output becomes more unreliable as these processes become increasingly influential on the overall mass balance. In other words, the reliability of the IV-MBM EQP v2.0 model output is expected to decrease as the overall persistence of the chemical in the test system decreases. The biotransformation capacities of a few cell lines have been investigated (e.g., primary hepatocytes, HepG2, HepaRG, [55,56,57]) but in general data for model parameterization are lacking. Also note that the overall persistence of the chemical in the test system is a function not just of a chemical’s susceptibility to abiotic (e.g., hydrolysis) and cell-mediated reactions but also the distribution of the chemical in the test system.

Changes to system characteristics and cellular parameters (e.g., proximate composition, growth) over time also cannot be explicitly included in IV-MBM EQP v2.0 calculations. The relevance of model limitations and uncertainties related to the equilibrium partitioning assumption can be assessed by applying time-variant distribution models (e.g., [17,22]) and a publication describing a such version of the IV-MBM model is forthcoming. Finally, although the IV-MBM EQP v2.0 model explicitly includes the phases typically deemed most relevant (e.g., headspace, serum lipid, serum albumin, vessel walls, cells), other phases such as other dissolved organics (e.g., amino acids), extracellular matrix (ECM), adhesive well plate coverings and vessel wall coatings (e.g., poly-d-lysine) may be important and warrant inclusion in some instances. Resources would have to be dedicated to quantifying these partitioning behaviors and then deriving reliable estimation approaches to facilitate their inclusion in the model.

The other critical factor limiting the application of the IV-MBM EQP v2.0 model (and all other in vitro distribution models) is the lack of data required for model parameterization. For example, cellular volumes and proximate compositions (i.e., lipid, protein, water content) have been published in the peer-reviewed literature for only a few of the many hundreds of cell lines used in in vitro testing (e.g., MCF7, HCT116, HEK293T, HepG2) [20,36,37]. In vitro tests using 3D cell configurations (e.g., spheroids) are becoming more common and their properties also need to be better characterized to support model parameterization. Similarly, with a few exceptions (e.g., DMEM), the compositions and sorption capacities of the various cell culture media used are not well characterized. Likewise, the potential variability in the lipid and protein content of sera added to in vitro systems across different species, batches, and commercial providers are unclear. Given that the equilibrium distribution of organic chemicals in in vitro test systems is essentially a function of the volume of each phase and its sorption capacity, these data gaps represent a challenge to the application of these models for quantitative IVIVE. With respect to sorption to plastic, the comparisons between the spLFERs given in the Supplementary Material (Section S2) demonstrate that differences in predicted partition ratios can sometimes occur e.g., for neutral organics with *log K_OW_* from 2–4 and *log K_OW_* > 7.5 and may influence model performance. While these spLFERs are derived from chemicals covering a reasonable range of partitioning properties, the data sets are relatively small, and additional measurements would still be valuable (irrespective of the model evaluations presented herein).

Although many aspects of the model parameterization mentioned above can be addressed by relatively simple measurements, there still needs to be a coordinated and systematic effort to generate, compile and publish such data. Although routine, these measurements are needed to support the quantitative extrapolation of in vitro test data. Characterization and standardization of test protocols for in vitro tests deemed regulatory priorities would be beneficial but will not do much to facilitate the further interpretation of the huge amount of in vitro data reported online and in the scientific literature using nominal concentrations. Concurrently, more systematic efforts to evaluate the performance of the available in vitro distribution models are necessary, particularly for predominantly charged IOCs. Further case studies for “non-volatile” and “volatile” neutral organics would also be valuable to increase confidence in available models and possibly support additional modifications to address issues identified during the empirical work and model evaluation exercise. This research effort will also require a substantial amount of coordination and funding but ultimately seems vital to facilitate the regulatory use and acceptance of the quantitative extrapolation of in vitro data for hazard assessment, risk assessment, and chemical management. The assessments and conclusions reached using the quantitative IVIVE of in vitro data can then be combined with other lines of evidence to support regulatory decision-making.

## Figures and Tables

**Table 1:** Summary of data sets used to evaluate the IV-MBM EQP v2.0 model.

Data Set	Number, Type of Chemicals, Range of *log K_OW,N_* and *log K_AW,N_*	Cell Type and Assay Conditions	Exposure Period (h)
Tanneberger et al. [38]	*n* = 27 Neutral (25) & IOC (2) *log K_OW,N_* = −4.15–7.54 *log K_AW,N_* = −10.5–0.31	Fish RTgill-W1 24 well plate 19 °C FBS = 0%	24
Dupraz et al. [39]	*n* = 13 Neutral organics *log K_OW,N_* = −3.20–5.50 *log K_AW,N_* = −13.3–−3.92	Algae 48 well plate 20 °C FBS = 0%	96
Huchthausen et al. [40]	*n* = 12 Neutral (1) and IOCs (11) *log K_OW,N_* = −0.07–3.97 *log K_AW,N_* = −17.6–−5.21	Human MCF7 and HEK293H 96 well plate 37 °C FBS = 10% (AREc32) FBS = 2% (PPARγ)	24
Schug et al. [41]	*n* = 16 Neutral organics *log K_OW,N_* = 1.83–6.25 *log K_AW,N_* = −5.19–−0.69	Fish RTgutGC 24 well plate 19 °C FBS = 0%	24

Chemicals are classified as IOCs if an ionizable functional group is present.

**Table 2:** Model performance (Factor of Agreement, *FoA*) of the IV-MBM EQP v2.0 using Tanneberger et al. [38] dataset under various assumptions regarding the effective volume of headspace (HS multiplier) and sorption to plastic (spLFER).

IV-MBM EQP v2.0 Model Assumptions	*FoA* 10*^MB^*	*FoA* 10*^MAE^*	r^2^
“Non-volatiles only” (*log K_AW_* or *log D_AW_* < −4.0)			
Fischer et al. plastic spLFER HS Multiplier = 1	0.84	1.27	0.81
Kramer plastic spLFER HS Multiplier = 1	0.96	1.21	0.83
All Chemicals			
Fischer et al. plastic spLFER HS Multiplier = 1	1.33	1.89	0.54
Fischer et al. plastic spLFER HS Multiplier = 10	1.17	1.67	0.71
Fischer et al. plastic spLFER HS Multiplier = 50	0.98	1.59	0.82
Fischer et al. plastic spLFER HS Multiplier = 100	0.88	1.65	0.86
	All Chemicals		
Kramer plastic spLFER HS Multiplier = 1	1.58	2.03	0.41
Kramer plastic spLFER HS Multiplier = 10	1.37	1.77	0.63
Kramer plastic spLFER HS Multiplier = 50	1.11	1.64	0.81
Kramer plastic spLFER HS Multiplier = 100	0.99	1.69	0.86

HS = Headspace, *MB* = Model bias (Equation (12)), *MAE* = Mean Absolute Error (Equation (13)).

**Table 3:** Number of compounds with “volatility issue”, “solubility issue” and predicted membrane concentrations (*C_MEM_*) consistent with baseline toxicity for DSL chemicals with *AC*50s in the ToxCast AEID 767 and AEID 1325 assays.

AEID	“Volatility Issue”	“Solubility Issue”	Predicted *C_MEM_* > 20 mM	Predicted *C_MEM_* > 60 mM
767	4/329	13/329	145/329	76/329
1325	1/217	14/217	86/217	60/217

## Data Availability

The Excel/VBA version of the IV-MBM Ver.2.0 model is freely available from https://arnotresearch.com/models/. The R/EAS-E Suite implementation can be freely accessed by registering for the online application at the following website: https://arnotresearch.com/eas-e-suite/. Requests for the computer code developed for both implementations of the IV-MBM Ver.2.0 model will be considered on an individual basis and subject to agreements regarding its further use or dissemination to other parties.

## Supplementary Materials

The following are available online at https://www.mdpi.com/article/10.3390/toxics9110315/s1, Section S1: Additional details on the IV-MBM EQP v2.0 tool, Section S2: Comparison of spLFERs for predicting sorption to plastic (*log K_PlW_*), Section S3: Blood-water and plasma-water partitioning, Section S4: Additional details on model evaluation data sets, Section S5: Additional details on illustrative model application. Section S6: Relationships between partitioning properties and empirical C24/C0 ratios for Schug et al., Section S7: IV-MBM EQP v2.0-Phys-Chem Inputs.
