# Supplementary material for: Update and Evaluation of a High-Throughput In Vitro Mass Balance Distribution Model: IV-MBM EQP v2.0
Source: Toxics. 2021 Nov 20;9(11):315. doi: 10.3390/toxics9110315 (PMC8625852; doi:10.3390/toxics9110315)
Supplement: Supplementary file 1 [file toxics-09-00315-s001.zip › toxics-1431533-supplementary_R2.pdf]

toxins

MDPI

Supplementary Materials: Update and Evaluation of a High-Throughput In Vitro Mass Balance Distribution Model: IV-MBM EQP v2.0

James M. Armitage, Alessandro Sangion, Rohan Parmar, Alexandra B. Looky and Jon A. Arnot

Section	Contents	Page
S1	Additional details on the IV-MBM EQP v2.0 tool	SI-1
S2	Comparison of spLFERs for predicting sorption to plastic (log K_{PIW})	SI-2
S3	Blood-water and plasma-water partitioning	SI-3
S4	Additional details on model evaluation data sets	SI-4
S5	Additional details on illustrative model application	SI-6
S6	Relationships between partitioning properties and empirical C24/C0 ratios for Schug et al.	SI-7
S7	IV-MBM EQP v2.0 - Phys-Chem Inputs	SI-8
	References	SI-8

Section S1. Additional details on the IV-MBM EQP v2.0 tool

1.1. Sorption to anionic phospholipids

Schmitt [1] noted that the sorption of basic ionizable organic chemicals (IOCs) to anionic phospholipids (e.g., phosphatidylserine) appears to be substantially greater than sorption to "neutral" phospholipids (e.g., phosphatidylcholine) while the opposite is likely for acidic IOCs due to the type of electrostatic interactions possible for the two phospholipid types.

The IV-MBM EQP v2.0 tool allows the user to specify the fraction of phospholipids in the cell that are anionic and then a proportionality constant which is used to relate the sorption capacity of anionic phospholipids to neutral phospholipids. The default fraction of anionic phospholipids in the cell is 0.175 and the default proportionality constants are 20 for basic IOCs and 0.05 for acidic IOCs. In other words, the partition ratio of a given chemical for anionic phospholipids is assumed to be 20-fold larger for bases and 20-fold smaller for acids relative to the partition ratio for neutral phospholipids. The overall membrane-water partitioning is then calculated using these inputs and the partitioning estimates for neutral phospholipids

1.2. Preliminary treatment of lysosomal sequestration

The IV-MBM EQP v2.0 tool allows the user to specify the fraction of the cell occupied by lysosomes, the median diameter (nm) of the lysosomes and the pH of the lysosomal fluid. The default fraction of cell occupied by lysosomes and diameter are 0.0068 and 500 nm. The default pH of the lysosomal fluid is 5.1. The enhancement to the sorption capacity of the cell for basic IOCs is then calculated from the pH difference (i.e., ion trapping multiplier) and the volume of cell occupied by lysosome and 'normal' cell constituents.

1.3. Estimating partitioning properties of neutral organics and IOCs

As described in the main text, the IV-MBM EQP v2.0 tool can be used to simulate IOCs using the minimal set of inputs which include molecular weight (MW), melting point (MP), IOC type (Acid or Base), pKa and octanol-water partition ratio of the neutral

form (log $K_{OW,N}$). In the absence of any other user-entered partitioning data, spLFERs are used to estimate the required biopartitioning data, i.e.,

Membrane-water partitioning

$$logK_{MW,N} = 1.01logK_{OW,N} + 0.12 \quad [2]$$

Serum albumin-water partitioning

If log $K_{OW,N} < 4.5$

$$logK_{SaW,N} = 1.08logK_{OW,N} - 0.70$$

If log $K_{OW,N} \geq 4.5$

$$logK_{SaW,N} = 0.37logK_{OW,N} + 2.56 \quad [3]$$

These spLFERs are applied to both neutral organics and the neutral form of IOCs. The partitioning properties of the charged form of the chemical is estimated using scaling factors i.e.,

$$\log K_{MW,I} = \log K_{MW,N} - dMW$$

Table S1. Scaling factors to relate the partitioning properties of neutral and charged forms of IOCs.

Property	Scaling Factor	Default for acidic IOCs	Default for basic IOCs
Octanol-water<br>DOM-water	dOW	3.1	3.1
Membrane-water	dMW	1.0	1.0
Serum albumin-water	dSaW	0.0	1.3
Plastic-water	dPlW	3.1	3.1

pH-dependent distribution ratios are then calculated following the standard procedures, e.g., see Schwarzenbach et al. [4].

See the Supporting Information of Armitage et al. [5] for details on other aspects of the IV-MBM EQP v2.0 tool related to temperature and salinity (ionic strength) adjusted property values.

Section S2. Comparison of spLFERs for predicting sorption to plastic (log K_{PlW})

The predicted plastic-water partition ratios (log K_{PlW}) are plotted as a function of hydrophobicity (log K_{OW}) for the two spLFERs given in the main text (Eq 5, 6) in Figure S1. The spLFER derived by Fischer et al. [6] in units of m^3/m^2 using sorption data over a 48 h period rather than from data for the full experimental duration (400 h) is also included in Figure S1.

$$log\ K_{PlW} = 0.47\ logK_{OW,N} - 4.64 \quad (S1)$$

For ease of interpretation, the magnitude of difference between the predicted log K_{PlW} using equation 5 (Kramer) and 6 (Fischer et al., full sorption data) is plotted as a function of hydrophobicity in Figure S2. As shown in Figure S2, there is reasonable agreement between the spLFERs for chemicals with log K_{OW} from 4.0 – 7.5 (within a factor of approximately 5). There are large differences for hydrophilic chemicals (log K_{OW} < 2) but these differences are less of a concern because the predicted mass balance is not strongly influenced by sorption to plastic. The differences for chemicals with log K_{OW} from 2.0 to 4.0 and log K_{OW} > 7.5 are more of a concern because the influence of sorption to plastic on the predicted mass balance is greater. We are not currently able to recommend one spLFER over the other but do note that the Fischer et al. [6] spLFERs were derived using a larger and more diverse set of chemicals.

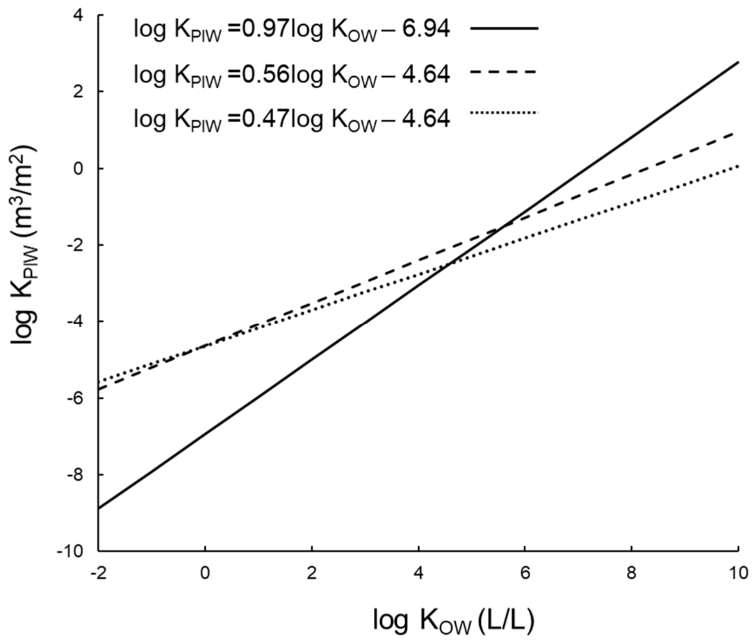

Figure S1. Predicted plastic-water partition ratios (log K_{PIW}) as a function of hydrophobicity (log K_{OW}) for the three spLFERs

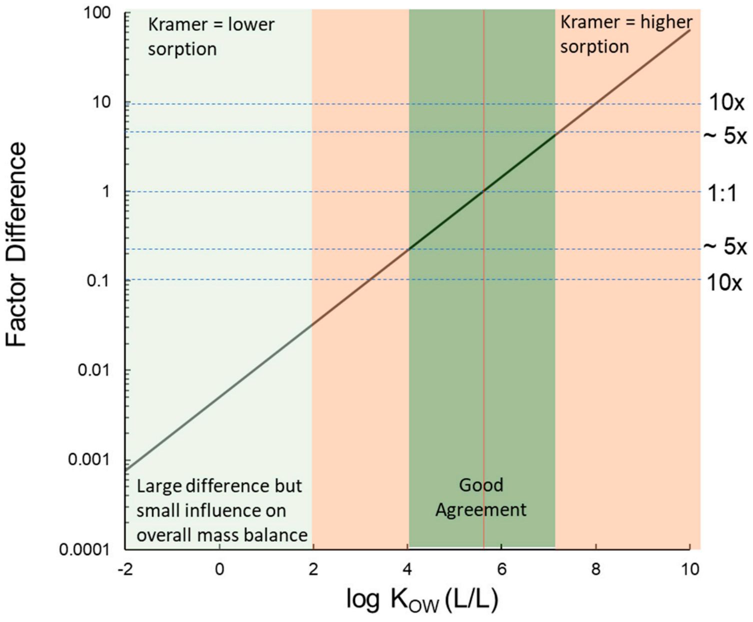

Figure S2. Magnitude of difference between the Kramer and Fischer et al. spLFER as a function of hydrophobicity (log K_{OW})

Section S3. Blood-water and plasma-water partitioning

Blood-water and plasma-water partition or distribution ratios are calculated using partitioning information and the proximate composition of blood and plasma suggested by Endo et al. [7]. Note that these calculations are intended for extrapolation to humans and so the partitioning data are adjusted to pH 7.4, 37 °C and 0.15 M regardless of the assay conditions.

Table S2. Proximate composition of blood and plasma assumed by the IV-MBM EQP v2.0 tool.

Phase	Storage lipid	Membrane lipid	Bulk protein	Serum albumin	Water
Blood	0.0013	0.0022	0.16	0.016	0.8205
Plasma	0.0015	0.0008	0.015	0.029	0.9537

Section S4. Additional information on model evaluation data sets

The selected physical-chemical properties and nominal doses are summarized in the Excel spreadsheet accompanying the Supporting Information. We also recommend that readers consult the original publications [8–11]. Other key input parameters are summarized in the following tables.

Table S3. Key input parameters for the Tanneberger et al. [8] simulations.

Input Parameter	Selected Value
Well plate characteristics	
Well plate size	24
Well diameter	15.6 mm
Growth area	190 mm^2
Total well volume	3400 μL
Volume of medium added	2000 μL
Medium characteristics	
Temperature	19 ^{o}C
pH	7.4
Ionic strength	0.15 M
FBS volume fraction (%)	0.00
Cell characteristics	
Cell seeding density	300 000
Mass per cell	0.525 ng
Total mass of cells	0.158 mg
Storage lipid content	0.01
Membrane lipid content	0.04
Protein content	0.055
Water content	0.895

Table S4. Key input parameters for the Dupraz et al. [9] simulations.

Input Parameter	Selected Value
Well plate characteristics	
Well plate size	48
Well diameter	11 mm
Growth area	95 mm^2
Total well volume	1700 μL
Volume of medium added	1000 μL
Medium characteristics	
Temperature	19 ^{o}C
pH	7.4
Ionic strength	0.15 M
FBS volume fraction (%)	0.00
Cell characteristics	
Cell seeding density	20000
Mass per cell	5.0 ng
Total mass of cells	0.1 mg
Storage lipid content	0.01
Membrane lipid content	0.04
Protein content	0.05

Water content	0.90

Table S5. Key input parameters for the Huchthausen et al. [10] simulations.

Input Parameter	Selected Value
Well plate characteristics	
Well plate size	96
Well diameter	6.4 mm
Growth area	32 mm^2
Total well volume	360 μL
Volume of medium added	220 μL
Medium characteristics	
Temperature	37 ^{o}C
pH	7.4
Ionic strength	0.15 M
FBS volume fraction (%) – AREc2	10
FBS volume fraction (%) – PPARγ	2.0
Cell characteristics – AREc2	
Cell seeding density	20000
Mass per cell	10 ng
Total mass of cells	0.2 mg
Storage lipid content	0.00046
Membrane lipid content	0.00414
Protein content	0.0514
Water content	0.944
Cell characteristics – PPARγ	
Cell seeding density	20000
Mass per cell	7.3 ng
Total mass of cells	0.146 mg
Storage lipid content	0.001
Membrane lipid content	0.009
Protein content	0.08
Water content	0.91

Table S6. Key input parameters for the Schug et al. [11] simulations.

Input Parameter	Selected Value
Well plate characteristics	
Well plate size	24
Well diameter	15.6 mm
Growth area	190 mm^2
Total well volume	3400 μL
Volume of medium added	2000 μL
Medium characteristics	
Temperature	19 oC
pH	7.4
Ionic strength	0.15 M
FBS volume fraction (%)	0.0
Cell characteristics	
Cell seeding density	120000
Mass per cell	1
Total mass of cells	0.12 mg
Storage lipid content	0.01
Membrane lipid content	0.04
Protein content	0.055
Water content	0.895

Section S5. Additional details on illustrative model application

The selected physical-chemical properties and nominal doses are summarized in the Excel spreadsheet accompanying the Supporting Information. See [12,13] for cell properties of the two cell lines (MCF7, HCT116)

Table S7. Key input parameters for the AEID767 simulations (MCF7 cell line).

Input Parameter	Selected Value
Well plate characteristics	
Well plate size	1536
Well diameter	1.63 mm
Growth area	2.5 mm^2
Total well volume	12.5 μL
Volume of medium added	5.0 μL
Medium characteristics	
Temperature	37 ^{o}C
pH	7.4
Ionic strength	0.15
FBS volume fraction (%)	10
Cell characteristics	
Cell seeding density	1500
Mass per cell	10 ng
Total mass of cells	0.015
Storage lipid content	0.00046
Membrane lipid content	0.00414
Protein content	0.0514
Water content	0.944

Table S8. Key input parameters for the AEID1325 simulations (HCT116 cell line).

Input Parameter	Selected Value
Well plate characteristics	
Well plate size	1536
Well diameter	1.63 mm
Growth area	2.5 mm^2
Total well volume	12.5 μL
Volume of medium added	5.0 μL
Medium characteristics	
Temperature	37 ^{o}C
pH	7.4
Ionic strength	0.15
FBS volume fraction (%)	10
Cell characteristics	
Cell seeding density	4000
Mass per cell	2.61 ng
Total mass of cells	0.01044 mg
Storage lipid content	0.00254
Membrane lipid content	0.02286
Protein content	0.12
Water content	0.8546

Section S6. Relationships between partitioning properties and empirical C24/C0 ratios for Schug et al.

The empirical C24/C0 ratios for the Schug et al. data set are presented as a function of log K_{OW} in Figure S3.

As can be seen in Figure S3, the empirical C24/C0 ratios for chemicals with similar reported partitioning properties can be substantially different. For example, the empirical C24/C0 ratios for chemicals with approximately the same log K_{OW} (~ 5.0) and log K_{AW} (~ -3.0) range from 0.30 to 0.74. On the other hand, Verdox (log K_{OW} 4.75, log K_{AW} -1.09) is reported to have an empirical C24/C0 ratio larger than Veloutone (log K_{OW} = 4.70, log K_{AW} = -1.49) even thought the opposite pattern is expected. Finally, the second most hydrophobic chemical Tonalide (log K_{OW} 5.70, log K_{AW} -3.19) has a reported empirical C24/C0 ratio 1.5-fold greater than several chemicals that are less hydrophobic (whereas the opposite is also expected). Volatilization out of the test system cannot explain these results given the log K_{AW} of these chemicals.

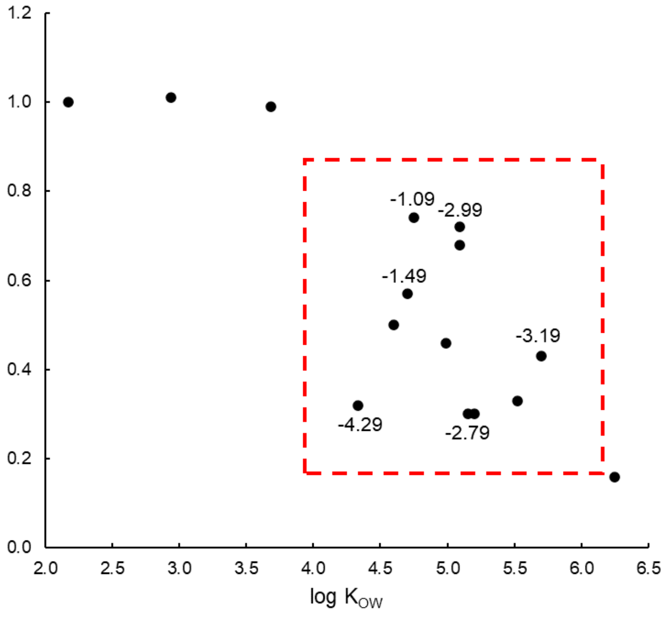

Figure S3. Empirical C24/C0 ratios for the Schug et al. data set as a function of log K_{OW}. Air-water partition coefficients (log K_{AW}) are given on the plot for select chemicals.

Section S7. IV-MBM EQP v2.0 - Phys-Chem Inputs

Provided as a separate Microsoft Excel® file:

IV-MBM EQP v2.0 - Phys-Chem Inputs.xlxs

References

1. Schmitt, W. General approach for the calculation of tissue to plasma partition coefficients. *Toxicol In Vitro* 2008, 22:457–467.
2. Endo, S.; Escher, B.I.; Goss, K.-U. Capacities of Membrane Lipids to Accumulate Neutral Organic Chemicals. *Environ. Sci. Technol.* **2011**, *45*, 5912–5921.
3. Endo, S.; Goss, K.-U. Serum Albumin Binding of Structurally Diverse Neutral Organic Compounds: Data and Models. *Chem. Res. Toxicol.* **2011**, *24*, 2293–2301.
4. Schwarzenbach, R.P.; Gschwend, P.M.; Imboden, D.M. *Environmental Organic Chemistry, 2nd Edition*. John Wiley & Sons, Inc.: Hoboken, NJ, USA, 2003.
5. Armitage, J.M.; Wania, F.; Arnot, J.A. Application of Mass Balance Models and the Chemical Activity Concept To Facilitate the Use of in Vitro Toxicity Data for Risk Assessment. *Environ. Sci. Technol.* **2014**, *48*, 9770–9779.

6. Fischer, F.C.; Cirpka, O.A.; Goss, K.-U.; Henneberger, L.; Escher, B.I. Application of Experimental Polystyrene Partition Constants and Diffusion Coefficients to Predict the Sorption of Neutral Organic Chemicals to Multiwell Plates in in Vivo and in Vitro Bioassays. *Environ. Sci. Technol.* **2018**, *52*, 13511–13522.
7. Endo, S.; Brown, T.N.; Goss, K.-U. General Model for Estimating Partition Coefficients to Organisms and Their Tissues Using the Biological Compositions and Polyparameter Linear Free Energy Relationships. *Environ. Sci. Technol.* **2013**, *47*, 6630–6639.
8. Tanneberger, K.; Knöbel, M.; Busser, F.J.M.; Sinnige, T.L.; Hermens, J.L.M.; Schirmer, K. Predicting Fish Acute Toxicity Using a Fish Gill Cell Line-Based Toxicity Assay. *Environ. Sci. Technol.* **2013**, *47*, 1110–1119.
9. Dupraz, V.; Stachowski-Haberkorn, S.; Wicquart, J.; Tapie, N.; Budzinski, H.; Akcha, F. Demonstrating the need for chemical exposure characterisation in a microplate test system: toxicity screening of sixteen pesticides on two marine microalgae. *Chemosphere* **2019**, *221*, 278–291.
10. Huchthausen, J.; Mühlenbrink, M.; König, M.; Escher, B.I.; Henneberger, L. Experimental Exposure Assessment of Ionizable Organic Chemicals in In Vitro Cell-Based Bioassays. *Chem. Res. Toxicol.* **2020**, *33*, 1845–1854.
11. Schug, H.; Maner, J.; Hülskamp, M.; Begnaud, F.; Debonneville, C.; Berthaud, F.; Gimeno, S.; Schirmer, K. Extending the concept of predicting fish acute toxicity in vitro to the intestinal cell line RTgutGC. *Altex* **2020**, *37*, 37–46.
12. Fischer, F.C.; Henneberger, L.; König, M.; Bittermann, K.; Linden, L.; Goss, K.-U.; Escher, B.I. Modeling Exposure in the Tox21 in Vitro Bioassays. *Chem. Res. Toxicol.* **2017**, *30*, 1197–1208.
13. Henneberger, L.; Mühlenbrink, M.; Fischer, F.C.; Escher, B.I. C18-Coated Solid-Phase Microextraction Fibers for the Quantification of Partitioning of Organic Acids to Proteins, Lipids, and Cells. *Chem. Res. Toxicol.* **2019**, *32*, 168–178.
